# Psychological impacts of AI-induced job displacement among Indian IT professionals: a Delphi-validated thematic analysis

**DOI:** 10.1080/17482631.2025.2556445

**Published:** 2025-09-02

**Authors:** Vinod Sharma, Saikat Deb, Yogesh Mahajan, Avishek Ghosal, Manohar Kapse

**Affiliations:** a Symbiosis Centre for Management and Human Resource Development (SCMHRD), Symbiosis International (Deemed University), Pune, India; b Business Analytics, Jaipuria Institute of Management - Indore Campus, Indore, India

**Keywords:** Artificial intelligence, job displacement, mental health, technostress, IT professionals, qualitative research, India, organizational psychology

## Abstract

**Purpose:**

This study investigates the psychological impact of Artificial Intelligence (AI)-driven job displacement among Indian IT professionals. It specifically explores how individuals psychologically experience the loss of roles due to automation, and how these experiences influence their emotional, cognitive, and behavioural well-being.

**Method:**

A qualitative phenomenological approach was used to capture the lived experiences of 24 IT professionals who faced AI-induced job loss or reassignment. Data were collected via in-depth semi-structured interviews and analysed through thematic analysis. To ensure rigour and theoretical saturation, a three-round Delphi process involving 20 domain experts—spanning clinical psychology, organizational behaviour, and AI policy—was used to validate and refine the emergent themes.

**Results:**

Six core psychological themes were identified: emotional shock, erosion of professional identity, chronic anxiety and anticipatory rumination, social withdrawal, adaptive and maladaptive coping strategies, and perceived organizational betrayal. These themes reflect a multilayered resource loss, including identity, control, employability, and social belonging.

**Conclusion:**

AI-driven role redundancy in the Indian IT sector is more than a labour market shift a deep psychological disruption. This study underscores the urgent need for organizations, mental health practitioners, and policymakers to develop anticipatory and compassionate interventions that can buffer the mental health consequences of technological transformation.

## Introduction

The ground beneath Today’s workforce is shifting at an unprecedented pace. The rapid ascent of artificial intelligence (AI), machine learning, and automation is not merely transforming industries; it fundamentally redefines the nature of work. This transformation is now more evident than in high-skill, high-volume sectors such as information technology (IT), where intelligent systems are increasingly handling tasks that once demanded deep expertise.

India, home to one of the world’s largest and most dynamic IT sectors—with approximately 5.43 million professionals contributing to a segment that generated US$254 billion in FY 2024 (NASSCOM, 2024)—is confronting significant disruption. Although resilient amid global volatility (Ministry of Finance, 2024), the swift integration of generative AI, robotic process automation, and algorithmic decision-making is generating increasingly complex challenges for the human workforce. Recent surveys indicate that 96% of Indian professionals use AI or generative AI tools in their work, and 94% perceive that mastering these technologies is essential for career progression; yet, many remain deeply concerned about potential job displacement if they do not upskill (Emeritus, 2025; Ministry of Finance; 2024). An IIM‑Ahmedabad study of white‑collar workers found that while 55% have adopted AI and 48% have received training, 68% fear their roles could be automated within five years (Chakrabarti et al., 2024). Complementing these findings, independent research on Indian IT firms’ HR practices confirms that although AI is enhancing operational efficiency and personalized employee development, it simultaneously raises concerns about workforce displacement and algorithmic dependence (Rawashdeh, 2025; Naik, 2024; Sharma et al., 2025). These developments underscore a precarious moment as technology outpaces established human capital strategies, thrusting professionals into uncertainty over job security and the enduring relevance of their skills.

The World Economic Forum (2023) projects that nearly 44% of workers’ core skills will change within the next five years, signifying an urgent need for reskilling and continuous learning. Indian IT giants, such as TCS, Infosys, and Wipro, have reportedly reduced their workforce by over 60,000 employees as part of a broader strategic realignment (Mandavia, 2024). In May 2023, estimates suggest that approximately 4,000 tech roles were lost to AI-related automation (Sengupta, 2023). Public statements by leaders, such as Zoho’s CEO Sridhar Vembu, have further highlighted the possibility that traditional software development roles may soon become obsolete (Times of India, 2025).

Globally, this picture is not sobering. The International Monetary Fund estimated that AI could affect 40% of jobs worldwide (Cazzaniga et al., 2024), and Goldman Sachs predicted that generative AI could replace tasks equivalent to 300 million full-time jobs (Hatzius et al., 2023). In India, growing awareness of these shifts is prompting large-scale upskilling efforts. LinkedIn’s 2024 Workforce Report (Baruah, 2024) notes that 94% of Indian firms are preparing to retrain their employees in response to AI disruption. Companies such as Microsoft and Deloitte invest heavily in AI infrastructure and human capital development (Soni & Sophia Mary, 2025; Business Standard, 2025). However, these investments coexist, with a noticeable slowdown in net hiring, as noted in the NASSCOM—BCG analysis (Bardia, 2024).

However, considerable attention has been devoted to the economic and operational dimensions of this transformation (Acemoglu & Restrepo, 2020). The psychological impact, particularly among those that are most directly affected, remains relatively underexplored. Preliminary data, such as the (American Psychological Association’s, 2023 *Work in America* survey, have indicated growing workplace stress in the face of technological change (American Psychological Association, 2023). In India, anecdotal evidence from HR circles and mental health forums (MyndStories, 2025) suggests increasing levels of anxiety, burnout, and a sense of disorientation among IT workers navigating AI-related role shifts or job loss.

There is a growing need to understand how AI-induced job displacement affects the mental health, identity, and coping mechanisms of affected professionals. Studies have shown that job loss is associated with emotional trauma, perceived loss of self-worth, and disrupted identity (Brand, 2015; Blustein & Guarino, 2020). However, AI-related displacement adds a unique layer of threat, as it questions not only the economic role of workers but also their relevance in a future job market increasingly dominated by machines (Susskind, 2020). Despite these challenges, little empirical work has examined how Indian IT professionals emotionally experience such transitions, what meaning they assign to them, and how they cope—creating a critical gap in both research and practice.

This study seeks to move beyond surface-level analyses of workplace stress. Our aim was to understand how AI-induced job disruption specifically affects Indian IT professionals at a psychological level. We anchor our inquiry into the Conservation of Resources (COR) theory (Hobfoll, 1989; Hobfoll et al., 2018) and the technostress framework (Tarafdar et al., 2007). Of particular interest are perceptions of resource loss, such as the erosion of professional identity, control, or skill relevance, and the emergence of “techno-stressors, ”such as techno-insecurity and techno-uncertainty.

## Research objectives

To explore these issues, we propose a qualitative investigation using phenomenological methods to document the lived experiences of Indian IT professionals who have undergone significant job changes or displacements due to AI integration. To enhance the depth and credibility of our analysis, we also employed the Delphi method to incorporate expert consensus from academia, industry, and mental health professionals.

The study’s core objectives are to:

1. Identify and categorize the key psychological stressors associated with AI-related job displacement or role modification among Indian IT professionals.
2. Validate these stressor categories through expert consultation using a Delphi method and explore their interconnections.
3. Develop a conceptual model to inform both organizational strategies and mental health interventions tailored to the needs of affected professionals.

This research contributes to the growing literature on the human dimensions of digital transformation by spotlighting demographic—skilled IT professionals whose vulnerabilities are often masked by the assumption of future readiness. The insights gained are expected to be relevant not only within the Indian context but also for global economies such as Australia, where similar transitions are underway.

## Literature review

### AI and workforce disruption

The integration of artificial intelligence (AI), machine learning (ML), and automation into organizational workflows has led to a significant transformation in job structures across industries. The IT sector, traditionally viewed as a growth engine, is now at the forefront of this disruption. Globally, AI has been shown to automate not only repetitive tasks, but also decision-making roles, posing risks to both low- and mid-skilled technical jobs (Brynjolfsson & McAfee, 2014; Chui et al., 2016). In the Indian context, a McKinsey report (Chui et al., 2016) projected that nearly 30% of current IT functions are at risk of automation by 2030, with an estimated 300,000 roles likely to be displaced in the next few years owing to AI integration.

While much of the existing literature addresses the economic and structural implications of AI, relatively less attention has been paid to its psychological consequences, particularly in emerging economies, such as India, where IT employment is central to middle-class stability and social mobility.

### Psychological impacts of job loss

Extensive occupational psychology literature has demonstrated that involuntary job loss is strongly correlated with a range of adverse mental health outcomes, including depression, anxiety, somatic symptoms, and suicidal ideation (Paul & Moser, 2009; Wanberg, 2012). Unemployment often entails a loss of income, status, social networks, and purpose factors critical to psychological well-being (Jahoda, 1982). Recent studies have further shown that technology-induced job displacement may lead to higher levels of psychological distress compared to traditional layoffs, owing to the perceived permanence and inevitability of automation (Kellogg et al., 2020; Makridakis, 2017).

In India, job identity is closely tied to personal and familial pride, particularly in the IT sector, and is often associated with economic success and upward mobility. Job loss in this context may therefore trigger not only individual stress, but also interpersonal strain, social withdrawal, and internalized shame (Gupta & Sagar, 2022).

### Technostress and AI-induced anxiety

The concept of technostress, defined as psychological stress experienced by the introduction or presence of new technologies, has gained prominence in recent years (Tarafdar et al., 2007, 2024). While initial research focused on overwork, information overload, and digital fatigue, newer studies now link technostress to the fear of redundancy, inadequate reskilling, and cognitive overload due to constant upskilling demands (Rohwer et al., 2022).

A qualitative study by Calvard and Jeske (2018) found that employees in automation-threatened roles often exhibit learned helplessness and future anxiety, especially when organizational support structures are weak. In the Indian IT ecosystem, where large segments of the workforce rely on legacy skill sets, such as manual testing, L1 support, and data entry, the introduction of AI and robotic process automation (RPA) has accelerated these stressors (Bhatia, 2023).

### Theoretical perspectives on resource loss and coping

This study is grounded in the Conservation of Resources (COR) theory (Hobfoll, 1989; Hobfoll et al., 2018), which posits that psychological stress occurs when individuals experience loss or threat to valued resources (e.g., employment, self-efficacy, and status). AI-induced job loss represents not only financial insecurity, but also the symbolic loss of professional identity, autonomy, and future prospects. Additionally, Cognitive Appraisal Theory (Folkman, 2013; Lazarus, 1984) supports the notion that individuals’ perception of their coping ability—whether they see change as a challenge or threat—plays a key role in their psychological adjustment to displacement.

Moreover, research suggests that coping mechanisms vary widely: while some employees engage in proactive behaviours, such as reskilling and networking, others report emotional exhaustion, social withdrawal, and substance use as maladaptive responses (Blustein et al., 2013; El Khawli et al., 2022).

### Research gap

Although there is an expanding body of research on the economic and operational aspects of AI-driven workforce transitions, significantly fewer studies explore the psychological impacts of such disruptions—especially in the Indian context. A critical reading of the literature indicates that existing studies in India have largely centred on reskilling strategies, labour market shifts, and organizational adaptation (NASSCOM, 2024, Deloitte, 2024), while the emotional and cognitive toll of job displacement remains underrepresented. For instance, recent reports by the Ministry of Finance (2024) highlight growing anxiety among workers regarding AI adoption, yet these sources stop short of examining individual psychological outcomes. Furthermore, peer-reviewed empirical studies combining mental health, automation, and job identity loss in the Indian IT sector are notably limited. This thematic and methodological underrepresentation suggests a critical gap, which this study addresses by offering an in-depth qualitative inquiry into how Indian IT professionals experience and make sense of AI-induced job disruption. This study aims to fill this gap by using a phenomenological qualitative design and triangulated expert validation (Delphi method) to identify, categorize, and conceptualize the psychological effects of AI-driven job displacement in the Indian IT sector.

This study is based on two fundamental theories: the Conservation of Resources (COR) theory and the Technostress framework. Stress, in accordance with the COR theory (Hobfoll, 1989), means that when people are threatened with losing or actually lose resources that they value, such as employment, skills, self-efficacy, and more, these resources are the central ones in the identity of professional IT. This model illuminates the affective and cognitive cost of job loss to AI as a major resource loss in AI numbers.

Moreover, we use the Technostress approach, which focuses inter alia on the stress that people may feel when confronted with the request and challenge of a new technology (Tarafdar et al., 2007). In the context of our research, this model enables analysis of the manner in which the perceived threat of AI, with associated job loss, results in a novel type of technological stress, or “techno-insecurity”, which is evidenced through psychological and emotional suffering. These are the two theories in light of which we “read” our data, although references are also made to other theoretical ideas, for instance, Identity Theory (Stryker & Burke, 2000) and Cognitive Appraisal Theory, in so much as they enable us to discuss how practitioners understood their engagement.

## Methodology

### Research design and theoretical framework

To understand the psychological impacts of artificial intelligence (AI) and automation induced job displacement on Indian IT professionals, this research used a qualitative phenomenological research methodology. Based on a constructivist-interpretivist lens, with the purpose of exploring how affected individuals make subjective sense of and respond to job loss, role redundancy, or enforced reskilling in the face of rapid technological advancement, this study was conducted. Phenomenology was considered a preferred qualitative methodology because it is a powerful tool for capturing vivid experiences, particularly the emotional and existential phenomena (e.g., anxiety, identity loss, uncertainty) (Moustakas, 1994). Drawing upon participants’ idiographic accounts, the exploration eyes shedding light on more fine-grained psychological reactions to a novel, relatively uncharted work stressor, AI-triggered job uncertainty.

Following Moustakas (1994) transcendental phenomenology, the research team engaged in epoche (bracketing) by reflecting on and documenting their prior assumptions about AI-related job loss through reflexive journaling before data collection. This process was crucial in minimizing interpretive bias. Phenomenological reduction was practiced during coding by focusing solely on participants’ descriptions, setting aside theoretical interpretation until after initial theme development. Imaginative variation was applied during the Delphi validation phase, where alternative meanings and structural interpretations were explored collaboratively with domain experts to better understand the essential structure of psychological distress related to displacement.

### Participant recruitment and sample characteristics

Participants were recruited through purposive and snowballing sampling from India’s four major IT hubs: Bengaluru, Hyderabad, Pune, and Gurugram. Eligibility criteria included age between 25 and 45 years, current or recent employment in the IT sector, experience of job loss, extended bench time without active projects, and redeployment to lower-skill roles resulting from organizational automation strategies. The sample consisted of 24 participants balanced equally by gender (12 males and 12 females) and included professionals such as software developers, DevOps engineers, backend and frontend developers, UI/UX specialists, and IT support staff, roles commonly impacted by AI interventions such as robotic process automation (RPA), generative AI tools, and machine-learning-driven testing systems.

While purposive and snowball sampling were used to identify participants with relevant displacement experience, we acknowledge that this approach may have yielded a relatively homogenous sample in terms of urban, professional, and educational backgrounds. The absence of participants from Tier-2 cities or rural IT hubs may limit the representativeness of experiences across India’s broader digital workforce. The study focused on IT professionals based in Tier-1 cities due to the higher density of tech employment and layoffs reported in these regions. However, we recognize that this urban focus may limit the transferability of findings to professionals in Tier-2 or emerging digital clusters, whose experiences may differ based on access to support systems and skill development infrastructure.

### Data collection procedures

Data collection involved in-depth semi-structured interviews conducted via secure video-conferencing platforms. Each interview lasted approximately 45 to 60 minutes and followed an interview guide developed based on the Conservation of Resources (COR) theory (Hobfoll, 1989) and technostress literature (Tarafdar et al., 2007, 2024). The interview questions focused on participants’ emotional reactions to displacement, perceptions of self-worth and future prospects, coping mechanisms, and experiences with organizational or institutional support. The interviews were conducted in both English and Hindi, with informed written consent, and transcribed verbatim. Data collection was continued until thematic saturation was achieved.

Interviews followed a semi-structured guide informed by COR theory and technostress literature. Sample questions included: “Can you describe how you felt emotionally after learning about your job displacement?” and “What changes did you experience in your self-perception or identity after the role change?” The guide was pilot-tested with three professionals and refined for clarity. Interviews were conducted in either English or Hindi, based on participant preference. Hindi interviews were transcribed and translated by bilingual researchers and cross-verified for semantic accuracy to maintain translation fidelity. Any culturally embedded terms were annotated with contextual explanations during coding.

### Delphi panel composition

The Delphi validation involved 20 domain experts selected based on their professional background and relevance to the study. This included:

- 6 clinical psychologists (5 Indian, 1 international)
- 5 organizational behaviour scholars from Indian universities
- 4 HR leaders from top-tier IT firms
- 3 mental health professionals specializing in occupational stress
- 2 AI ethics researchers
  All experts had 10+ years of experience in their respective domains. 18 out of 20 were Indian nationals.

### Consensus measurement criteria

Consensus was measured using a Likert-scale voting format (1 = strongly disagree, 5 = strongly agree). Themes with a minimum of 80% of respondents rating them ≥ 4 (agree/strongly agree) were retained. Themes failing to reach 80% in the first round were refined and re-evaluated in subsequent rounds.

## Validation metrics

To enhance rigour, inter-rater reliability was assessed during thematic coding using Cohen’s Kappa, with an agreement score of 0.82, indicating substantial agreement. This was computed using a randomly selected 20% subset of the transcripts, coded independently by two researchers.

### Delphi process flow

- **Round 1**: Presentation of 8 initial themes from thematic analysis
- **Round 2**: Modified themes based on expert feedback
- **Round 3**: Final validation and subtheme refinement
  Feedback was both quantitative (via Likert ratings) and qualitative (open-ended suggestions).

### Data analysis methodology

As outlined by Braun and Clarke (2006) thematic analysis was employed to analyse the data through six iterative steps: familiarization with transcripts, generating initial codes, searching for themes, reviewing and refining themes, defining and naming themes, and synthesizing findings into a coherent narrative. NVivo 12 software was used to systematically manage and organize the data. The coding process utilized both inductive and deductive approaches, and initial themes emerged from participants’ narratives and were later interpreted through the theoretical lenses of COR theory and technostress frameworks. Thematic saturation was monitored iteratively using NVivo logs. By the 20th interview, no new primary codes were emerging, and by the 22nd interview, only minor subthemes were added. Two additional interviews were conducted to confirm the redundancy of themes, confirming saturation. An audit trail of code development and thematic decisions was maintained through memos and timestamps within NVivo, ensuring transparency in the analytic process.

Thematic analysis was conducted following the widely recognized six-phase framework by Braun and Clarke (2006). This approach allowed for a flexible yet rigorous method to identify, analyse, and report patterns within the data. The process unfolded as follows:

1. **Familiarization with the data**: The researchers returned to and fully absorbed the data by repeatedly reading and rereading interview transcripts to fully understand the participants’ lived experiences. This involved the writing of notes and initial observations.
2. **Generating initial codes**: We performed a line-by-line coding of the transcripts. This was a primarily inductive process, with codes emerging directly from the data (e.g., “fear of replacement,” “feeling obsolete,” “financial stress”).
3. **Searching for themes**: We then reviewed the initial codes and collated them into potential themes, looking for patterns and recurring ideas. This phase involved both an inductive approach (allowing new themes to emerge) and a deductive approach (using the COR and technostress frameworks to organize codes).
4. **Reviewing themes**: The potential themes were then reviewed against the entire dataset to ensure they accurately reflected the meanings within the data. This involved an iterative process of refining the themes.
5. **Defining and naming themes**: Each theme was clearly defined and given a concise name to capture its essence and its relationship to the other themes.
6. **Producing the report**: The final step involved writing a detailed narrative of the themes, using rich, verbatim quotes from the participants to support our interpretations.

Additionally, we enhanced the methodological rigour through a three-round Delphi method. After the initial thematic analysis, the researchers, along with a panel of experts, reviewed the emergent themes to validate their accuracy and relevance, thereby adding an extra layer of trustworthiness to our findings.

### Validation through the Delphi method

To enhance the credibility and robustness of the findings, a modified Delphi method was implemented, involving three rounds of review with 20 domain experts. These experts included clinical psychologists, organizational psychologists, HR managers from IT firms, and academic researchers specializing in work psychology and AI policies. They reviewed and provided feedback on emerging thematic constructs. Themes and subthemes were refined based on consensus, defined as at least 80% agreement across rounds. The Delphi process serves as an external validation mechanism, contributing to the conceptual integrity of the final thematic model.

### Ethical considerations

Ethical approval was obtained from the Academic Integrity Committee of Symbiosis Centre for Management & Human Resource Development, Pune, India [reference no: SCMHRD/2024/102]. This study was conducted in accordance with the ethical principles outlined in the Declaration of Helsinki. The participants provided informed written consent and were assured of confidentiality and the right to withdraw from the study at any time. Pseudonyms were used to protect participant anonymity, and all data were securely stored in encrypted password-protected systems.

### Ensuring research rigour and trustworthiness

This study maintained rigour by applying Lincoln and Guba’s (1985) criteria for trustworthiness. Credibility was strengthened through member checking and triangulation through the Delphi process. Transferability was supported by providing detailed descriptions of the study context and participant profiles. Dependability and confirmability were addressed through a detailed audit of coding decisions and reflexive journaling throughout the research process. Collectively, these strategies reinforced the methodological integrity and psychological relevance of the study, ensuring that its findings were both grounded and theoretically meaningful.

## Results

A thematic analysis of interviews with 24 displaced IT professionals revealed a complex interplay of emotional, cognitive, and behavioural responses to job loss triggered by AI and automation. Six psychological themes emerged from the data, each comprising multiple interrelated subthemes. These themes were validated through a three-round Delphi process involving 20 domain experts to ensure conceptual clarity and practical relevance. The following section presents each theme in detail, accompanied by illustrative quotes (pseudonyms used) and supported by expert consensus. An overview of these emergent psychological themes, their subthemes, phenomenological essence, and representative participant responses is presented in Table I.

**Table I.** Thematic summary with phenomenological interview extracts.

Theme	Subthemes	Phenomenological Essence	Verbatim Extracts (Participant Quotes)
1. Psychological Shock and Emotional Volatility	- Initial disbelief - Emotional outbursts	Sudden emotional destabilization triggered by unexpected job loss.	“It felt surreal—like the ground just vanished beneath me.” (35-year-old male Software Developer) “One day I was presenting a sprint review, the next day I was obsolete.” (29 years old female Scrum Master)
2. Loss of Professional Identity and Self-Worth	- Feeling outdated - Erosion of confidence and relevance	Internalized sense of obsolescence and diminished self-efficacy.	“I built backend systems for 10 years. Now they say a bot does it faster. What am I then?” (38 years old female Systems Analyst) “They didn’t just take the job; they took the part of me that mattered.” (42 years old male Data Administrator)
3. Anxiety, Rumination, and Future Uncertainty	- Anticipatory anxiety - Health issues	Persistent worry over employability, relevance, and future roles.	“Every notification from LinkedIn is like a panic trigger now.” (30 years old female UI/UX Designer) “Even when I sleep, I wake up thinking—what if I’m never needed again?” (34 years old female QA Lead)
4. Disengagement and Social Withdrawal	- Avoidance of peers and platforms - Isolation	Withdrawal from social/professional networks due to shame or comparison anxiety.	“I muted all my office WhatsApp groups. Couldn’t take the constant updates from people still ‘in’.” (33 years old female DevOps Engineer) “I stopped checking LinkedIn. Everyone’s skilling up; I was barely coping.” (37 years old male Tech Support)
5. Coping Responses (Adaptive and Maladaptive)	- Mindfulness, reskilling (adaptive) - Bingeing, substance use (maladaptive)	Mixed coping strategies ranging from proactive reskilling to avoidant behaviors.	“I joined a cloud certification course—it gave me a sense of direction.” (27 years old female Frontend Developer) “Mostly, I drank and binge-watched. I just didn’t want to think.” (29 years old male QA Engineer)
6. Organisational Betrayal and Institutional Apathy	- Loss of trust in employers - Inadequate external support	Sense of abandonment by employers and lack of empathetic support.	“They said AI would assist us. But it replaced us. And no one even talked about mental health.” (41-year-old female IT Support Manager) “The career counselling helpline kept sending me PDFs—nothing personal or helpful.” (36-year-old male Backend Developer)

### Psychological shock and emotional volatility

For many individuals, the experience of losing a job due to AI was not just unexpected; it was disorienting. The ground appeared to have shifted overnight. This emotional issue can be understood through trauma theory, particularly Janoff-Bulman’s (Olson, 1994) notion of shattered assumptions, which argues that when core beliefs about safety, fairness, or control are not taken care of, people enter a state of psychological crisis. Several participants reported feelings of shock and disorientation, highlighting that job loss was experienced not merely as the end of employment but as a profound disruption to their sense of self and reality. It was not only the occupational role that was gone, but also a part of their perceived life and personal identity.

In contrast, while some of the themes—psychological shock, identity loss, organizational betrayal—are interrelated, they are analytically distinct phases and aspects of the displacement experience. Mental shock is the first emotional disorganization after role loss. Our translation bundle reduction also serves as the index for the lower-order process that erodes identity—the cognitive tearing of professional self-concept across time. Organizational betrayal represents the perceived lack of trust in relationships between employees and employers and a lack of care and concern within the institution.

We mapped these not as isolated entities but as sequential and interacting layers of psychological disruption.

Nearly all the participants reported experiencing acute emotional reactions following displacement. The most frequently cited responses were shock, disbelief, anger, and helplessness. For many, job loss was not anticipated, as AI-related displacement was perceived as abstract or distant until it directly affected them.

I had just received a project excellence award a month before. Then I was told, ‘Your role is no longer needed.’ It felt surreal—like the ground just vanished. (35-year-old male software developer)

Participants described a rapid shift from confusion to emotional volatility, marked by outbursts, insomnia, and feelings of betrayal, particularly in cases where employers had previously reassured them of job security. This theme received full consensus (100%) from expert reviewers regarding its centrality to the displacement experience.

### Loss of professional identity and self-worth

Many participants struggled not just with unemployment but with what it meant for their identity. For years, their work has shaped how they see themselves and how others perceive them. The sudden erosion of this identity, especially when framed by the narrative that AI could now do their job better or faster, felt deeply personal. This experience resonates with identity theory (Stryker & Burke, 2000), which explains how roles like “software engineer” or “project lead” become embedded in our self-concept. As Ashforth and Kreiner (1999) noted, even difficult jobs contribute to a person’s sense of meaning. When such roles are made redundant, people often feel as though they have been made redundant.

Job displacement due to AI was experienced not just as a career disruption but also as an erosion of personal identity. The participants expressed a profound sense of diminished self-worth and relevance in a rapidly evolving ecosystem. The sense of identity loss was especially pronounced among those who had built careers around now-obsolete skill sets such as manual testing or rule-based programming.

I was the go-to guy for backend architecture. Now I’m just a name in the ‘bench’ list. It’s not just a job—they’ve taken my confidence. (38-year-old female systems analyst)

The internalization of obsolescence, where participants perceived themselves as outdated or redundant, emerged as a critical subtheme. This theme resonated strongly with clinical psychologists in the Delphi panel, who linked it to markers of depressive cognition and negative self-appraisal.

### Anxiety, rumination, and fear of the future

The participants often described a persistent state of worry that did not switch off, even at night. Many mentioned checking LinkedIn with a sense of dread or waking up anxious about their futures. These are classic signs of anticipatory anxiety, which Beck (1979) linked to negative thought patterns that spiral out of control. Rando’s (1986) work on anticipatory grief is also useful here: some participants seemed to mourn not just what they had lost but what they feared would never come back. Lazarus and Folkman’s (1984), transactional model of stress helps frame this further; when people feel that they lack the resources or control to manage change, it often results in chronic emotional distress.

Participants commonly described persistent anxiety marked by repetitive thoughts about financial insecurity, skill irrelevance, and employability. This theme manifested as both anticipatory (fear of what is next) and generalized (across multiple life domains) anxiety. A subset of participants reported panic attacks, somatic symptoms (e.g., chest tightness and headaches), and changes in appetite or sleep patterns.

Every time I open LinkedIn, I get a knot in my stomach. Someone posts about AI this or automation that—and I feel like I’m disappearing from the future. (30-year-old female UI/UX designer)

Experts in the Delphi panel noted that the anxiety reported by participants resembled career-related existential stress with potential spillover effects on family dynamics, financial decision-making, and social functioning.

### Disengagement and social withdrawal

Many individuals pulled away from their social and professional circles—not out of anger but out of shame, fatigue, or emotional overload. Some avoided conversations with peers who were still employed or muted notifications from groups that reminded them of what they had lost. This type of withdrawal aligns with Festinger’s (1954) social comparison theory, in which upward comparisons lead to feelings of inadequacy. Gallie et al. (2003) also highlighted how unemployment can fuel the cycles of isolation and self-stigma. Over time, this can become a maladaptive form of coping, particularly when support networks are crucial for recovery.

A significant proportion of participants reported withdrawing from social and professional networks. Some discontinued communication with their former colleagues or avoided family discussions about employment. Social media avoidance, particularly platforms like LinkedIn, was common, reflecting both shame and fear of comparison.

I turned off notifications from all my work WhatsApp groups. I didn’t want to explain why I was jobless, especially when others were posting certificates from some AI course. (33-year-old female DevOps engineer)

Delphi experts flagged this theme as a potential early sign of depressive disengagement, especially among participants who showed reduced motivation to seek re-employment or engage in upskilling activities.

### Coping responses: adaptive and maladaptive

Faced with emotional strain and uncertainty, people respond differently. Some turn inward, whereas others take action. A few participants found the purpose of reskilling or mindfulness routines, while others admitted to binge-watching or drinking to numb discomfort. Carver et al. (1989) suggested that people engage in either problem-focused strategies, which address the source of stress, or emotion-focused strategies, which manage the feelings it causes. Both responses were visible here, reflecting the human tendency to vacillate between resilience and retreat, depending on available support, mental stamina, and prior experience.

Participants employed a range of coping strategies following displacement. These were categorized as:

#### Adaptive coping

Proactive behaviours aimed at regaining control, such as enrolling in online certifications, practicing mindfulness, engaging in freelancing, and maintaining daily routines. For instance, one participant noted, *“The cloud certification helped me stay focused. I needed to feel relevant again.”*

#### Maladaptive coping

Avoidance-based or psychologically harmful responses, such as excessive alcohol consumption, binge-watching, emotional withdrawal, and reliance on sleep aids. As shared by a male QA engineer, *“For the first two months, I binge-watched shows all day. I felt numb. It was like I didn’t know how to be productive without deadlines.” (29-year-old male QA engineer)*

This distinction was based on functional outcomes—whether coping mechanisms supported or hindered psychological adjustment.

### Perceptions of organisational betrayal and institutional apathy

One of the most painful threads running through the interviews was the feeling of being let down, not just by employers but by the larger system. Many participants said they were promised support, retraining, or at least transparency but felt discarded and dismissed. According to Rousseau (1995), the concept of psychological contracts helps explain this sense of betrayal; when implicit promises between workers and organizations are broken, the emotional fallout can be intense. Greenberg (1990) extended this to organizational justice, while Litz et al. (2009) frame such experiences as moral injuries, especially when people feel that their dignity has been undermined.

Many participants voiced resentment towards employers, describing the displacement as poorly managed, lacking transparency, and lacking psychological support. There was a shared perception that organizations prioritized cost saving and technological efficiency over employee well-being.

They spoke about ‘upskilling’ in town halls, but the next day my access card didn’t work. No warning, no dignity.

(41-year-old female IT support manager)

**Comparative Insights: Gender and Role-Based Patterns:** While core themes were consistent across participants, important differences emerged across gender and job role lines:

- **Gender Differences**: Female participants more frequently described internalized shame, loss of confidence, and heightened fear of irrelevance. This was particularly salient in roles such as DevOps and backend engineering, where visibility and male dominance were perceived as barriers. As one participant shared, *“I kept thinking maybe I just wasn’t as sharp as the others. Maybe I had missed the AI wave.”*
- **Role-Based Variation**: Professionals in support and QA roles expressed a stronger sense of disposability. In contrast, those in development or design roles reported greater motivation for proactive upskilling. For example, a 29-year-old female UI/UX designer said, *“I knew I had to pivot—design thinking isn’t easily automated, but I had to reframe my skills.”*

**Contextual Variation and Intersectionality**: The psychological impact of displacement was shaped by contextual factors, including age, years of experience, city, and access to resources:

- **Age & Experience**: Older professionals (35–45) often articulated fears around long-term irrelevance and age bias. A male backend developer noted, *“After 15 years in this field, I don’t know where else I fit. They want young coders who are AI-native.”*
- **City & Ecosystem**: Participants from cities like Bengaluru and Pune, with denser skilling ecosystems, reported more access to re-employment or retraining resources. Those in Gurugram or Hyderabad expressed greater helplessness and institutional apathy.

These contextual differences highlight the intersectional nature of psychological vulnerability—shaped not just by displacement but by the interaction of social location, support systems, and perceived future agency.

Several respondents also expressed disappointment with institutional systems, such as job portals, counselling hotlines, or skilling platforms, which they described as generic or inaccessible. Delphi reviewers noted that this sense of betrayal may complicate re-employment efforts by eroding organizational trust. The credibility and relevance of each identified theme were supported by expert consensus, as summarized in Table II.

**Table II.** Delphi panel validation of mental health challenge themes.

Mental Health Challenge (Theme)	Experts Endorsing Theme (n = 20)	Consensus Level (%)	Status	Summary of Expert Comments
Psychological Shock and Emotional Volatility	20/20	100%	Accepted	Strong consensus that initial emotional dysregulation is typical and clinically relevant.
Loss of Professional Identity and Self-Worth	19/20	95%	Accepted	Particularly salient in tech industries, aligns with theories of occupational identity loss.
Anxiety, Rumination, and Fear of the Future	20/20	100%	Accepted	Recognised as a chronic psychological risk in automation-related displacement.
Disengagement and Social Withdrawal	18/20	90%	Accepted	Validated as a behavioural indicator of emotional strain; some suggested deeper social analysis.
Coping Responses: Adaptive and Maladaptive	19/20	95%	Accepted	Experts recommended highlighting tech-induced coping fatigue.
Perceived Organisational Betrayal and Institutional Apathy	17/20	85%	Accepted with minor revisions	Suggested a clearer distinction between perceived betrayal and structural/systemic failures.

### Overview of thematic structure

The final thematic map consisted of six major themes and 14 subthemes, all of which reached ≥ 80% agreement across the three Delphi rounds. The themes collectively capture a trajectory of emotional dislocation, identity threat, chronic uncertainty, and coping variability situated within the broader context of technological transformation and institutional inadequacy. These findings offer a foundation for developing psychological support interventions tailored to the AI-displaced workforce in India and similar emerging markets.

## Discussion

This study examined the psychological consequences of AI-driven job displacement among Indian IT professionals using a phenomenological approach with expert validation using the Delphi method. The findings reveal a multilayered disruption affecting not only the participants’ employment status but also their emotional stability, identity coherence, and future orientation. The psychological disruption experienced by displaced IT professionals aligns with the Conservation of Resources (COR) theory (Hobfoll, 1989), but our data suggest a more layered loss profile. For example, the initial emotional volatility described by participants, *“I went numb for three days, couldn’t even tell my wife”*, reflects the sudden depletion of both emotional and identity-related resources. Rather than merely restating COR’s premise, this highlights how emotional shock and identity erosion co-occur as primary indicators of resource loss. Similarly, while technostress theory (Tarafdar et al., 2007) explains the cognitive overload due to rapid tech transitions, our data reveal that distress was not solely tech-related. It was also existential—participants felt not just under-skilled but “undesirable,” suggesting that displacement anxiety transcends skill obsolescence and enters the realm of symbolic disempowerment. Psychological contract theory (Rousseau, 1995) was invoked by participants when describing employer behaviour: *“I gave them 11 years, and they gave me one day’s notice.”* However, this wasn’t just about fairness—it invoked a moral rupture, reinforcing the theme of organizational betrayal. The data thus expand the psychological contract construct to include moral and emotional dissonance, not just transactional breach.

Our findings support the notion that AI-induced displacement triggers layered psychological disruptions, beginning with acute shock, followed by identity erosion and compounded by perceptions of organizational betrayal (Rawashdeh, 2025). These stages are sequential yet overlapping, consistent with models of resource loss and identity disintegration in crisis contexts (Ashforth & Kreiner, 1999; Hobfoll, 1989). Additionally, gendered and role-based distinctions illustrate how psychological fallout is not monolithic. Female professionals and support staff experienced deeper identity threats and reduced coping efficacy—challenging assumptions that tech workers are uniformly resilient. These findings align with research on occupational stratification and mental health vulnerability in fast-changing economies. To maintain interpretive clarity, we have revised paragraphs that previously blurred boundaries between social withdrawal, identity crisis, and organizational justice. These constructs are now presented as sequential psychological reactions, rather than simultaneous. For instance, social withdrawal often preceded identity disturbance, while perceived organizational injustice intensified it later.

The initial emotional responses of shock, disbelief, and anger, as captured in Theme 1, represent an acute stress reaction consistent with the “alarm phase” of COR theory, wherein individuals confront the sudden depletion of key resources (Halbesleben et al., 2014). Participants’ descriptions of emotional volatility mirror prior research on involuntary job loss (Paul & Moser, **2009), but** with an added layer of technological unpredictability, underscoring the unique psychological uncertainty posed by AI. Unlike the traditional layoffs triggered by economic downturns, displacement in this context was seen as part of a permanent systemic shift, contributing to sustained emotional dysregulation.

Theme 2, relating to the loss of professional identity and self-worth, reflects the symbolic resource loss described in COR theory. Participants felt personally invalidated, often equating redundancy to personal failure. This aligns with prior findings that job loss affects not only economic security but also disrupts core psychological structures such as competence, purpose, and belonging (Blustein et al., 2013; Jahoda, 1982). In the Indian IT context, where employment in technology is strongly associated with upward mobility and social capital, the threat to one’s professional identity appears especially destabilizing. These experiences also intersect with constructs in occupational identity theory, which posits that one’s sense of self is often deeply entwined with one’s professional roles (Ashforth & Kreiner, 1999).

Theme 3, involving persistent anxiety and future-oriented rumination, is indicative of the chronic stress phase described in COR literature. Participants articulated concerns about employability, technological irrelevance, and inability to meet familial or societal expectations—psychological phenomena that align with anticipatory and technostress (Tarafdar et al., 2007). In several cases, these concerns translate into somatic symptoms and functional impairments, highlighting the need for psychological assessment and early intervention in displaced populations.

The findings also reveal patterns of social withdrawal (Theme 4), which serve as both a consequence and a coping mechanism. The avoidance of social media and professional networks—spaces once associated with status and competence—suggests internalization of shame and perceived inferiority. This is consistent with prior studies showing that job loss may result in reduced social participation and self-imposed isolation, which in turn amplifies mental health risks (Brand, 2015). The emotional distancing reported by the participants also reflects the erosion of social support, a critical secondary resource in COR theory, further compounding psychological vulnerability.

Theme 5 illustrates the bifurcation of coping strategies, with some participants engaging in adaptive behaviours, such as upskilling and mindfulness, while others resorted to maladaptive strategies, such as binge-watching, substance use, or emotional disengagement. This divergence aligns with transactional models of stress and coping (Lazarus & Folkman, 1984), where individuals’ appraisal of controllability influences their coping style. Participants who perceived agency in their situation were more likely to adopt problem-focused strategies, whereas those overwhelmed by uncertainty leaned towards avoidant or palliative coping. Importantly, the presence or absence of organizational support systems and family encouragement appeared to mediate coping behaviours.

Theme 6, perceived organizational betrayal and institutional apathy, emerged as a critical moderator of psychological distress. Many participants expressed dissatisfaction and moral outrage in an impersonal and opaque manner in which the layoffs were executed. This aligns with the literature on psychological contract violation (Morrison & Robinson, 1997), which links broken employer promises with emotional exhaustion, loss of trust, and reduced engagement. Additionally, the lack of targeted, empathetic mental health resources from institutional bodies underscores the systemic gaps in India’s digital work infrastructure, particularly when navigating mass-scale job transitions driven by AI.

While the majority of participants reported distress, a notable minority exhibited psychological resilience and adaptive coping. These individuals reframed displacement as an opportunity for reinvention. One backend developer explained, *“Honestly, this pushed me to finally pursue the data science track. I was scared, but now I feel more relevant than ever.”*

Such accounts point to the role of personal agency, self-efficacy, and social capital as buffering factors. These counter-narratives challenge a monolithic view of job displacement and align with post-traumatic growth literature (Tedeschi & Calhoun, 2004), where individuals may emerge stronger after adverse events.

Collectively, these findings extend the existing research on unemployment, technostress, and occupational psychology by introducing a technology-specific displacement model grounded in the experiences of workers in an emerging economy. This study contributes to a growing but underdeveloped literature on the psychological toll of AI-induced job disruption, demonstrating that such impacts are not merely economic or skill-based but deeply personal and psychological. Furthermore, it highlights the urgent need for interdisciplinary intervention strategies involving organizational policies, mental health services, and social safety nets that can buffer the psychological challenges caused by rapid technological displacement.

Rather than simply applying existing theories, our findings suggest an integrative model where COR theory explains resource depletion, technostress theory helps interpret cognitive and behavioural overload, and psychological contract theory captures relational disillusionment. However, these frameworks need extension to account for identity rupture, moral betrayal, and growth under duress—as emergent patterns in technology-driven economies.

## Implications

### Theoretical implications

This study makes several important theoretical contributions to the fields of occupational psychology, stress theory, and the emerging literature on the psychological impacts of technological disruption. First, it extends the Conservation of Resources (COR) theory (Hobfoll, 1989) into the domain of AI-driven employment displacement, showing how resource loss in this context is not limited to material deprivation but includes symbolic and psychological resources, such as identity, relevance, and perceived control. The findings confirm that the psychological threat posed by AI is not merely anticipatory but deeply experiential, affecting emotional regulation, self-worth, and behavioural adaptation.

Second, this study contributes to the technostress literature (Tarafdar et al., 2007) by introducing a qualitatively grounded view of technostress as a career-level phenomenon. Whereas previous studies have focused primarily on digital overload or ICT-related fatigue, this study identifies existential stressors rooted in automation anxiety, algorithmic replacement, and identity disruption, offering new pathways for theorizing technostress in the age of artificial intelligence.

Third, the findings reinforce the relevance of occupational identity theory (Ashforth & Kreiner, 1999) and psychological contract theory (Morrison & Robinson, 1997), especially in emerging economies, where job roles are deeply tied to social mobility, family expectations, and self-worth. The breakdown of the psychological contract, when combined with opaque organizational communication and inadequate support, leads to a cascade of emotional and cognitive strains. This offers a fertile area for theory development on trust erosion, employer-employee dynamics, and post-displacement recovery trajectories.

Finally, this study highlights the value of phenomenological inquiry in psychological research on technology and work, demonstrating the depth and nuance that narrative-based approaches can bring to understanding rapidly evolving, emotionally charged work transitions.

### Practical implications

From a practical standpoint, this study provides a timely and actionable set of recommendations for organizations, mental health professionals, and policymakers, particularly in the context of fast-changing technology-driven economies, such as India. A summary of the key psychological concerns and corresponding interventions across stakeholder groups is presented in Table III.

**Table III.** Practical implications of AI-Induced job displacement among Indian it professionals.

Stakeholder Group	Key Psychological Issues Identified	Recommended Interventions/Actions
Organisations & HR Managers	- Emotional shock and anxiety - Loss of trust - Identity crisis	- Transparent communication during automation rollouts - Embed Employee Assistance Programs (EAPs) - Introduce mental health first-aiders and transition coaches - Offer pre-layoff psychological preparedness sessions
Mental Health Practitioners	- Anxiety, rumination, and social withdrawal - Maladaptive coping	- Offer tailored CBT/ACT-based counselling for displaced professionals - Develop support groups for tech workers - Design digital mental health toolkits specific to job displacement scenarios
Policy Makers/Government	- Lack of institutional support - Underserved mental health needs	- Integrate psychological resilience into skilling missions (e.g., Skill India) - Mandate minimum mental health support in restructuring laws - Fund AI-displacement mental health hotlines and mobile clinics
Training Institutions/EdTech	- Inadequate coping readiness - Fear of irrelevance	- Embed future-of-work resilience modules in tech education - Incorporate emotional intelligence and coping skill training - Provide psychological mentoring as part of re-skilling/upskilling programs
Career Services/Job Platforms	- Stigma and identity loss - Disengagement from career identity	- Create AI-displacement-sensitive job portals - Develop career reorientation programs with embedded psychological support - Use inclusive messaging to reduce shame and reframe job transitions

#### Organisational practice and HR policy

IT firms and digital service providers must proactively address the psychological impacts of automation by implementing transparent communication strategies, reskilling programs with emotional scaffolding, and well-being frameworks. Mental health support must be integrated into redundancy management processes through structured interventions such as Employee Assistance Programs (EAPs), grief counselling, and transition coaching. To ensure early psychological intervention, HR managers should be trained to recognize signs of disengagement, burnout, and anxiety during role transitions.

#### Mental health interventions

Clinical psychologists and counselling practitioners must develop and offer specialized therapeutic frameworks tailored to job displacement through automation. Techniques such as cognitive behavioural therapy (CBT) and acceptance and commitment therapy (ACT) can be adapted to help affected individuals reframe job loss, manage uncertainty, and build psychological flexibility. Community-based mental health outreach and digital counselling platforms should be scaled to reach tech workers in second-tier cities, where access to in-person services may be limited.

#### Government and policymaking

Policymakers in India must proactively address the psychological impact of technological displacement. As automation and AI reshape the workplace, workers are increasingly vulnerable to stress, anxiety, and uncertainty about the future (Chakrabarti et al., 2024). Mental well-being must therefore become a foundational element of digital transformation strategies. Programs like the Skill India Mission should move beyond technical training to incorporate modules on psychological preparedness, peer mentoring, and resilience-building. The UK’s Skills for Jobs framework already recognizes resilience and mental health as core employability skills (UK Department for Education, 2022), offering a model India can adapt. Currently, India lacks formal pilot projects in this area, signalling a gap in integrating mental health into skilling ecosystems. Furthermore, there is an urgent need to legislate minimum standards of psychological support during transitions brought about by automation and organizational restructuring.

#### Career services and education providers

Universities and technical training institutes must update their career services and curricula to include future-of-work literacy, with an emphasis on emotional resilience, career adaptability, and identity flexibility. Embedding discussions on AI ethics, human-machine collaboration, and life after layoffs in technical education can help prepare the workforce for inevitable disruptions.

## Conclusion

This study provides a timely and nuanced understanding of the psychological toll of AI-induced job displacement in India’s rapidly evolving IT sector. Through phenomenological inquiry and expert-validated thematic analysis, the findings revealed that the experience of automation-related job loss is deeply personal and psychologically complex, marked by emotional shock, identity erosion, future-oriented anxiety, social withdrawal, and varied coping responses. Participants’ narratives underscore that the impact of technological disruption extends far beyond economic concerns, penetrating the core aspects of self-worth, purpose, and psychological security.

By interpreting these findings through the lenses of the Conservation of Resources (COR) theory and the technostress framework, this study contributes to the growing theoretical discourse on the future of work and its mental health implications. It also expands the existing models of occupational stress by demonstrating how AI-related displacement can lead to symbolic and existential resource loss, potentially triggering long-term psychological distress.

Practically, the research offers actionable insights for multiple stakeholders—employers, clinicians, policymakers, and educational institutions—underscoring the need for integrated, compassionate, and anticipatory strategies to support workers navigating technological transitions. The study advocates the inclusion of mental health provisions in organizational restructuring protocols, targeted therapeutic interventions for displaced professionals, and broader policy frameworks that protect psychological well-being in digital economies.

Given the accelerating pace of AI adoption, future research should build on these findings by employing longitudinal and mixed-methods approaches to track the psychological adaptation trajectories of displaced workers. Comparative studies across industries, job roles, and national contexts would further enrich our understanding of how technological change intersects human resilience and vulnerability. Because we stand at the intersection of technological innovation and human well-being, there is an urgent need to ensure that the future of work is not only efficient but also psychologically sustainable.

## Supplementary Material

Mental_Health_Fallout_of_IT_Professionals_with_Authors.docx

## Data Availability

Owing to the sensitive nature of the qualitative interview data and confidentiality agreements with participants, full transcripts cannot be publicly shared. De-identified excerpts or analytic codes can be provided upon reasonable request and subject to ethical approval.
